# Exploring the Mechanisms of Influence on COVID-19 Preventive Behaviors in China’s Social Media Users

**DOI:** 10.3390/ijerph17238766

**Published:** 2020-11-25

**Authors:** Zeyu Liu, Huijun Geng, Hao Chen, Meng Zhu, Tingshao Zhu

**Affiliations:** 1 Institute of Psychology, Chinese Academy of Sciences, Beijing 100101, China; liuzy@psych.ac.cn; 2 Department of Psychology, University of Chinese Academy of Sciences, Beijing 100049, China; 3 School of Law, China University of Political Science and Law, Beijing 102249, China; 2016811096@cupl.edu.cn; 4 Department of Social Psychology, School of Zhou Enlai School of Government, Nankai University, Tianjin 300350, China; hull1919@gmail.com; 5 Institute for Advanced Studies in Finance and Economics, HuBei University of Economics, Wuhan 430205, China

**Keywords:** COVID-19, preventive behaviors, influence mechanisms, behavioral immune system, social media

## Abstract

The outbreak of Coronavirus Disease 2019 (COVID-19) posed a powerful threat to human life. The preventive behaviors of individuals (e.g., home quarantine, disinfection, and wearing masks) play a key role in preserving and controlling the disease. In this case, as a motivational psychological system oriented toward avoiding infection, the behavioral immune system (BIS) may be activated and link to preventive behaviors. This study investigated the mechanisms through which emotional and cognitive processes resulted by BIS have promoted preventive behaviors in relation to COVID-19. We collected data on 22,005 active Sina Weibo users from 31 December 2019 to 8 February 2020 to measure their emotions (including disgust, happiness, and fear), cultural values (individualism and collectivism), moral concern (including purity vice, fairness vice, and authority virtue), and behavioral intentions (including isolation intention, protection intention, and aid intention) using Text Mind software and related dictionaries. Multiple regression and mediation analyses were performed to explore the relationships among variables. The results showed seven complete mediation paths (such as disgust–purity vice–protection intention). Each of these paths describes the effects of cognitive processes caused by BIS on preventive behaviors. We inferred that there may be path mechanisms such as disgust–cognitive processes–preventive behaviors. Using these results, policy makers can take appropriate measures to intervene in preventive behaviors (e.g., by posting disgusting images on social media to evoke disgust). The results can be used to explain differences in preventive behaviors among populations even in the face of similar thread levels. Furthermore, our research provides empirical evidence for the hypothesis of pathogen prevalence.

## 1. Introduction

The outbreak of Coronavirus Disease 2019 (COVID-19) led to a powerful threat to human life. The preventive behaviors of individuals (e.g., home quarantine, disinfection, and wearing masks) play a key role in preserving and controlling the disease [1,2,3]. However, many have refused to adopt preventive behaviors [4,5,6]. Additional study to promote individuals’ preventive behaviors is urgently needed.

Psychological factors have been shown to influence behaviors (e.g., self-concept and consumer behavior [7], or cultural values and conflict-handling behaviors [8]), including preventive behaviors. Previous research has found that infectious disease threat has led to many psychological changes, such as changes to mental health [9,10,11], risk perception [12], and moral judgment [13,14]. Thus, exploration of the psychological factors that change due to infectious disease influence preventive behaviors.

Mechanisms of influence from emotions (e.g., fear [15] or positive emotions [12]), risk perception [16], perception of social norms [17], perceived efficacy [12], and personality [18] have been found. For example, Wise, Zbozinek, Michelini, and Hagan [19] found that risk perception had a positive impact on preventive behaviors during the first week of the COVID-19 outbreak in the US. Social media exposure had an indirect positive effect on preventive behaviors, which were mediated by fear and anger [20]. However, risk perception and other psychological factors are general, and changes in these do not specifically relate to any disease in particular. For this reason, the promotion effect of these factors on preventive behaviors may be limited.

The behavioral immune system (BIS) is a motivational psychological system oriented toward avoiding infection [14]. As a product of evolution, the BIS can be activated by any environmental cues indicating infection [14], helping people avoid potential infection risk. Disgust and cognitive processes that facilitate the identification of potential pathogen sources and encourage avoidance behaviors are the main consequences of activation of the BIS [21,22,23,24]. Recent studies [23,25,26] have noticed the unique role of BIS and showed BIS linked to COVID-19 preventive responses. However, these research studies mainly focus on the influence of individual differences in the BIS on preventive behaviors, yet few studies how preventive behaviors were mediated by disgust and cognitive processes.

Traditionally, self-report questionnaires are used to measure emotion and other psychological factors. However, in the context of the COVID-19 outbreak, this approach is cumbersome, requiring additional implementation time and additional extra burden on participants [9]. Furthermore, a long-term questionnaire investigation may incur practice effects [27], producing biased results.

Many studies have shown that through analyzing contents expressed on social media, it is possible to identify emotions and attitudes and to infer people’s actual behaviors. For example, during H1N1, using Twitter data, Signorini, Segre, and Polgreen [28] tracked the rapid evolution of public sentiment and measured actual disease-related activity. Song, Song, An, Hayman, and Woo [29] found that search volumes for keywords related to suicide were predictive for suicide rates in South Korea. It has also been found that the usage frequency of social media increased greatly during the COVID-19 pandemic [30,31], which provided excellent conditions for the study of psychological characteristics using social media data. Sina Weibo is a leading social media platform in China with more than 550 million monthly active users in March 2020.

**Figure 1:**
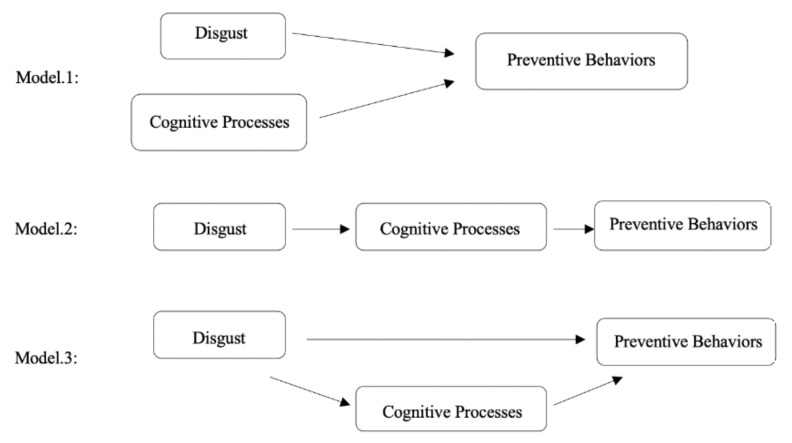
Hypothesized models of preventive behaviors.

Using Sina Weibo data, this study explored how the BIS activated by COVID-19 influenced preventive behaviors. To investigate the relationships among emotional results (disgust), cognitive results, and preventive behaviors, we proposed three hypotheses (Figure 1). The first asserts that disgust and cognitive processes are isomorphic [32], in that they have same impact on preventive behaviors (Model 1). Alternatively, disgust may play a causal role [33]. Therefore, there may be mechanisms that move along the disgust—cognitive processes—preventive behaviors pathway. However, it is not known whether disgust directly influences preventive behaviors (Models 2 and Model 3).

## 2. Materials and Methods

### 2.1. Participants and Data Collection

The samples in this study were drawn from a Sina Weibo data pool [34] that contains information relating to more than 1.16 million active users. The data retrieved included the users’ profiles, network behaviors, and posts. In this study, user privacy was closely protected, and the ethical principles reference stated by Kosinski, Matz, Gosling, Popov, and Stillwell [35] were strictly followed. The Ethics Committee’s approval code was H15009.

On 31 December 2019, the Wuhan Municipal government announced the COVID-19 epidemic and closed Huanan seafood market [31]. On 8 February 2020, the Central People’s Government of China issued a circular to allow work and production to resume [36]. In addition, the total new confirmed cases (not including Hubei province) had showed a downward trend before 8 February, indicating that the outbreak in China is basically under control. Therefore, we took 31 December 2019 to 8 February 2020 for our observation period, including the time when the outbreak in China was at its most serious and when people’s psychological traits were most influenced.

From the data pool, we selected Sina Weibo users that met the following criteria: had made at least one original post per day on average during the observation period, non-institutional authentication type, and regional authentication in mainland China rather than overseas, other, Hong Kong, Macau, or Taiwan. Our final sample was 22,005 active Sina Weibo users in 31 provinces. The demographic characteristics were as follows (Table 1). To illustrate that the sample is representative, we made a comparison with demographic characteristics of all users in our database.

### 2.2. Measurement of Psychological Traits and Procedures

Disgust was the emotional result of activation of the BIS. Previous studies found moral concern [37] and individualism/collectivism [38] were some of the cognitive results of activation of the BIS. For example, paying more attention to purity moral helped people avoid potential infection sources [39]. Collectivism emphasized in-group vigilance, which was more likely to promote protection against epidemics [40,41]. The interpretation of these psychological factors was as follows (Table 2). We employed Text Mind to measure these psychological traits.

Text Mind, developed by the Computational Cyber Psychology Lab, Institute of Psychology, Chinese Academy of Sciences, is a Chinese-language system for psychological analysis [42], including a Chinese word-segmentation tool and psychoanalytic dictionaries. This system first segmented users’ original Sina Weibo posts, extracted independent words, and linguistically labeled them [43]. Then, it used related dictionaries to determine semantic word-frequency statistics [44]. The specific keywords frequencies were used as the scores of disgust and other psychological traits. Three relevant dictionaries were used: Weibo Five Basic Moods Lexicon [45], Moral Foundations Dictionary (Chinese version) [46], and Dictionary of Individualism and Collectivism [47]. The reliability and validity of these dictionaries has been shown in many studies [45,46,47,48]. Details on these dictionaries are given in Table 3.

Individuals’ preventive behaviors were difficult to measure directly. Many unrelated factors may have affected these behaviors. For example, the purchase and use of masks during the observation period would have depended on mask production and distribution. Additionally, it was not safe for us to conduct a field survey.

Behavioral intention describes the state of preparation before individuals take action or perform behaviors [49]. It has been proven that the intention to perform preventive behaviors has significant and direct effects on behaviors to prevent COVID-19 spread [50]. Therefore, preventive behavioral intention is a good measurement of preventive behaviors. On the other hand, the content and style of text expression are the psychological projection of users’ emotion, cognitive style, and behavioral intention [51]. It is possible to measure behavioral intention from analysis of social media posts. Researchers usually measure behavioral intention by identifying relevant keywords found in social media posts [29,52,53]. We performed the following steps to select keywords related to behaviors to prevent COVID-19: select keywords relevant to COVID-19, select the most frequently used of these, and create dimensions in relation to semantics. We ultimately acquired five behavioral intentions (Table 4). The measurement of preventive behavioral intentions was the same as disgust.

## 3. Analysis and Results

The raw data were saved in an File S2. To balance the individual differences, all data were averaged by date. SPSS 26.0 and R 4.0.2 were used to conduct statistical analysis.

### 3.1. Identify Variables Related to COVID-19

There were six indicators representing COVID-19′s prevalence and threat level: total confirmed cases, total new confirmed cases, total suspected cases, total new suspected cases, total death cases, and total new death cases. Variables related to COVID-19 should be predicted significantly by any one of them. However, there may be collinearity in these indicators. To avoid unnecessary calculations, the correlation coefficients among these indicators were first calculated. Relevant data were taken from The National Health Commission of the People’s Republic of China [54]. Since the missing value was caused by the absence of new cases, we used 0 to replace the missing value. Results are shown in Table 5.

The correlation coefficients were all greater than 0.8. Therefore, we used total confirmed cases to predict psychological variables. Given the high inter-correlations, the other variables would have been similarly relevant.

The results (File S3) showed that the total confirmed cases could significantly predict emotion variables (except fear), collectivism/individualism, behavioral intentions (except dispelling rumors intention), fairness vice, purity vice, in-group vice, and authority virtue. We excluded variables not mentioned above in the following analysis.

### 3.2. Test the Relationship among Variables

Although we had proposed that there may be mechanisms of disgust–cognitive processes–preventive behaviors, we did not know the relationship among the sub-variables of different type variables.

#### 3.2.1. Emotions and Cognitive Processes

We first calculated the correlation coefficients between emotions and moral concern/cultural values. Results are depicted as follows (Table 6).

Correlation coefficients reveal associations between variables. Then, we conducted multiple regression analysis. Individualism et al. six variables (the first row of Table 6) were dependent variables, while the emotions significantly related to them were independent variables. Emotions other than disgust were control variables. If the standardized regression coefficient of independent variables was significant, we kept it; otherwise, we removed it from the model and reconstructed the model with the rest of the variables. We repeated this process until the standardized coefficients of independent variables in the model were all significant. Since the variance inflation factors (VIF) of emotions were greater than 1.2 [55], there was multicollinearity. Accordingly, we adapted ridge estimation instead of the ordinary least square (OLS) method. The results are shown in Table 7 (see File S4 for the results of processes).

Disgust could significantly explain individualism and the other dependent variables.

#### 3.2.2. Emotions and Behavioral Intentions

We performed the same process on emotions and behavioral intentions (Table 8 and Table 9, see File S4 for the results of processes).

Disgust could significantly explain isolation intention and anti-disease intention. Only happiness could predict the protection intention. Therefore, we could not refuse the hypothesis that disgust has no impact on protection intention in the case of controlling happiness and anger. However, the correlation between disgust and the protection intention was significant (*r* = 0.449, *p* < 0.001). Protective behaviors such as disinfection reduced the risk of infection more directly; they should have a closer relationship with BIS. Given this, we decided to retain the protection intention.

#### 3.2.3. Cognitive Processes and Behavioral Intentions

Since disgust was the core emotion of our study, we only retained variables that could be predicted by disgust after the two steps above. As we hypothesized, we took moral concern/cultural values as independent variables and behavioral intentions as dependent variables, repeating the same process. The results are listed in Table 10 and Table 11 (see File S4 for the results of processes).

According to the relationship among sub-variables, we planned to test the following paths: (1). disgust–purity vice–protection intention; (2). disgust–purity vice–anti-disease intention; (3). disgust–purity vice–isolation intention; (4). disgust–authority virtue–protection intention; (5). disgust–fairness vice–anti-disease intention; (6). disgust–fairness vice–isolation intention; (7). disgust–individualism–anti-disease intention.

### 3.3. Path Analysis

Mediation analysis was a path analysis used to assess potential causal mechanisms [56]. By calling package mediation in R, we tested the seven paths one by one. Table 12 shows the results of mediation analysis.

The seven paths we tested were all significant. Purity vice, fairness vice, authority virtue, and individualism were all full mediators. Disgust had no direct impact on behavioral intentions. The arousal of disgust first influenced moral concern and other cognitive processes caused by BIS and therefore preventive behaviors.

## 4. Discussion

As COVID-19 becomes more and more serious on a global level, studies of factors that promote preventive behaviors are becoming more important. This study collected posts from 22,005 Sina Weibo users from 31 December 2019 to 8 February 2020, and we measured the emotions, cultural values, moral concern, and behavioral intentions expressed in them using Text Mind software and related dictionaries. We conducted multiple regression and path analyses to explore the mechanisms of BIS in relation to preventive behaviors.

This study found mechanism pathways such as disgust—cognitive processes—preventive behaviors. In our results, purity vice fully mediated the first three paths. It was related to purity norms and reflected attention to the violation of purity norms [46]. Murray and Schaller [13] reported that the violation of purity always implies infection risk. Thus, people under its influence act in ways that preserve the sanctity of the body. Such actions include wearing masks, home quarantine, participating in the combat against COVID-19, and other behaviors help protect people from being contaminated by the virus.

Authority virtue, representing compliance with superiors’ orders, social norms, and rules [46], was a complete mediator in the fourth path. This suggests that during the COVID-19 pandemic, behaviors such as wearing masks and disinfecting became social norms in China.

The fifth and sixth path were fully mediated by the fairness vice. Fairness/cheating originates within two-way partnerships and helps produce win–win situations [37]. However, moral fairness was not only related to commodity exchanges: it also represented concern regarding interpersonal prejudices [37] (there are many words and phrases related prejudices in Moral Foundation Dictionary). Schaller and Neuberg [57] found that because pathogens cause physical changes, infectious disease threat can be expected to lead to prejudice against fat people, the elderly, and even those whose appearance deviates from the majority. Additionally, in our study, regional prejudices were also associated with this (e.g., discrimination against residents of the hardest-hit areas). Accordingly, people became more sensitive to those around them, resulting in a greater willingness to fight COVID-19. Meanwhile, they paid greater attention to whether people with such characteristics were quarantined.

Individualism is a full mediator for the final path. Freedom, independence, and self-concern were important contents for individualism [58]. During the outbreak, declines in individualism indicated that people were more concerned with cooperating with in-group members. Thus, the fight against COVID-19 was seen as a collective and nationwide struggle. Collectivism and individualism are two points in the same cultural dimension, but collectivism was not found to play the same role as individualism, which may be related to an aspect of our data: people express more about themselves on social media [59] than in other contexts (the value of individualism in our study was more often found than that of collectivism, but China is a typically collectivist country).

Our research provided empirical evidence for evolutionary hypotheses of pathogen prevalence. Previous studies on the BIS have been performed in the laboratory, where pathogen threat has been activated using images [46,60] and scent clues [61,62]. To the best of our knowledge, this is the first time that the BIS has been tested in a real epidemic situation. The total confirmed cases significantly explained disgust, individualism, and other psychological variables related to the BIS, indicating that changes in these variables could be caused by infectious disease threat. Meanwhile, change trends in the variables were consistent with the laboratory results. As a special affective state in the BIS, disgust significantly predicted individualism and six other variables. With the exception of the purity vice, all of the other variables were only explained by disgust. Protection intention, isolation intention and anti-disease intention were all avoidance behavioral intentions, while aid intention and dispelling rumors intention were not. Only avoidance behavioral intentions could be mediated by disgust and cognitive processes, which was consistent with the laboratory results that BIS encouraged avoidance behaviors.

Since the BIS is a special motivational system intended to avoid infection, the promotional effect of BIS on preventive behaviors may improve. A few brief suggestions for policy makers follow from our results. First, the significant role that disgust plays in preventive behaviors indicates that evoking disgust may be a sound strategy for promoting preventive behaviors. In fact, some scholars believe that showing attractive images of patients and viruses does not help contain COVID-19. People should instead confront disgusting images. Additionally, more studies using big data should be pursued to produce recommendations for psychological interventions. These are suitable for obtaining information on large groups and can avoid the risk of field interventions. As an example of such use, authorities could post disgusting images on social media and recommend disgusting news on the home page. Second, in the context of a sudden public health crisis, policy makers should pay greater attention to differences between populations. Our results indicate why differences are found in preventive behaviors among populations, even where the level of threat and disgust are similar. This may be related to cultural differences (e.g., between East and West [63]) or differences in moral concern (e.g., by social class [64]). Policy makers should take the most appropriate intervention in relation to the psychological characteristics of different groups.

Our study emphasizes a research transition in the use of the BIS from theory to application. Some implications for researchers can be found in this. First, we show that BIS can influence preventive behaviors, and we propose the influenced mechanisms. However, we only took cultural values and moral concern into consideration. Further investigation is required to determine whether other cognitive processes related to BIS play the same role as cultural values and moral concern. Our research may have some importance. It may contribute to promote preventive behaviors and may explain differences in preventive behaviors. In fact, it may help policy makers prepare before a sudden public health crisis, reducing the impact of such an epidemic on the public. Second, the emotion of disgust may have a unique value in this context. Although we were not able to make any causal inferences, our results provide evidence for Schaller’s arguments: disgust plays a causal role in a range of social and psychological phenomena. The arousal of disgust influences moral concern and other cognitive processes related to BIS and, ultimately, preventive behaviors. Research that more rigorously examines exactly when and how disgust is and is not causally implicated in the social psychological phenomena produced by the BIS will be useful [21].

There are some limitations. First, the dataset only contained data form 31 December 2019 to 8 February 2020. During this time, the outbreak in China was at its most serious. However, it was unknown whether our conclusions are applicable after the COVID-19 was basically under control. We would extend our data in the subsequent study to test our conclusions. Second, our analysis did not include demographic variables. In fact, this information was difficult to access. Not all users’ profiles were complete. Although we averaged all data by date in order to balance the individual differences, there may still be bias. Subsequent studies would control these variables to validate the conclusions. Third, our conclusions may have regional differences across mainland China. Future research would focus on looking for Wuhan-specific results. Finally, the study sample may be biased, as Sina Weibo users tend to be younger people living in urban areas.

For future research, we have some suggestions. Firstly, it is necessary to carry out similar research in European countries that are currently struggling with the epidemic. On the one hand, our results will benefit from such cross-cultural research. On the other hand, such studies will help these countries control COVID-19 better and more quickly. Secondly, according to present conclusions, further studies on interventions targeted at different populations are urgent. Such studies can reduce the costs of interventions and maximize the interventions effect.

## 5. Conclusions

In this study, we explored the mechanisms for psychological factors altered by the impact of infectious disease on preventive behaviors using social media big data. Our results showed the path disgust–cognitive–preventive behaviors. They also provided evidence for the argument that disgust played a causal role here. Using our results, policy makers can take appropriate measures to intervene in preventive behaviors.

## Figures and Tables

**Table 1:** Demographic characteristics of selected participants.

		Sample: n (%)	All Users: n (%)
Gender	Male	5803 (26.37)	452,887 (38.77)
	Female	16,202 (73.63)	715,274 (61.23)
Age	18–30	2364 (10.74)	149,994(12.84)
	30–40	1724 (7.83)	111,336 (9.53)
	40+	405 (1.84)	14,951 (1.28)
	Missing data	17,512 (79.58)	892,032 (76.36)
Region of location	Wuhan	374 (1.70)	17,061 (1.46)
	Other regions	21,631 (98.30)	1,151,095 (98.54)
Total		22,005 (100)	1,168,156 (100)

**Table 2:** The interpretation of moral concern and cultural values

	Dimension	Interpretation
Moral Concern [37]	Care/Harm	Related to care, attention, and protect others from harm, such as empathy and altruism
	Fairness/Cheating	Originated within two-way partnerships and helped to produce win–win situations
	Loyalty/Betrayal	In-group loyalty or betrayal
	Authority/Subversion	Complied with superiors’ orders, social norms, and rules
	Sanctity/Degradation	Related to preserve the sanctity of the body and spirit
Cultural Values [38]	Individualism	Freedom, independence, self-concern
	Collectivism	Solidarity, cooperation, interpersonal relationship

**Table 3:** Details on dictionaries used.

Dictionary	Dimension	Measured Psychological Trait	Example Words/Phrases ^1^
Weibo Five Basic Moods Lexicon	Happiness	Emotion of happiness	Carefree, High-spirited, Risus
	Disgust	Emotion of disgust	Filthy, Prostitutes, Shit
	Anger	Emotion of anger	Rage, Roar, NND ^2^
	Sadness	Emotion of sadness	Depressed, Cry, Melancholy
	Fear	Emotion of fear	Shiver, Terrified, Panic
Moral Foundations Dictionary	Harm Virtue/Vice	Care/Harm	Sympathy, Protection/Torment, Abuse
	Fairness Virtue/Vice	Fairness/Cheating,	Equal, Honest/Partisan, Prejudice
	Purity Virtue/Vice	Sanctity/Degradation	Noble, Clean/Lascivious, Trash
	Authority Virtue/Vice	Authority/Subversion	Comply, Duty/Illegal, Heresy
	In-group Virtue/Vice	Loyalty/Betrayal	Partner, Team/Liar, Let down
Dictionary of Individualism and Collectivism	Individualism	Individualism	I, You, Compete, Choose
	Collectivism	Collectivism	We, They, Cooperation, Sacrifice

^1^ All words or phrases are in Chinese (File S1). These are translated into English following their semantics. ^2^ A word usually used to insult others.

**Table 4:** Details of behavioral intentions.

Dimension	Interpretation	Example Words/Phrases
Protection Intention	Self-protection intention aimed to avoid infection	Disinfection, Washing hands
Isolation Intention	Intention to remain home voluntarily and travel less	Home quarantine, Voluntary isolation
Anti-disease Intention	Intention to participate in the fight against COVID-19	Battle against COVID-19, Fighting COVID-19
Aid Intention	Assistance intention to hardest-hit areas	Donation, Aid, Assistance
Dispelling Rumors Intention	Denial and correction intention of rumors	Not believing in, spreading, or making up rumors

**Table 5:** Correlation coefficients among six indicators.

Variable	1	2	3	4	5	6
1. Total confirmed cases	1					
2. Total new confirmed cases	0.920 ***	1				
3. Total suspected cases	0.966 ***	0.974 ***	1			
4. Total new suspected cases	0.815 ***	0.932 ***	0.928 ***	1		
5. Total death cases	0.999 ***	0.917 ***	0.966 ***	0.820 ***	1	
6. Total new death cases	0.972 ***	0.966 ***	0.991 ***	0.917 ***	0.975 ***	1

*** *p* < 0.001.

**Table 6:** Correlation coefficients between emotions and moral concern/cultural values.

Emotion	Individualism	Collectivism	Fairness Vice	In-Group Vice	Authority Virtue	Purity Vice
Happiness	0.525 ***	−0.571 ***	−0.453 ***	−0.261	−0.514 ***	−0.813 ***
Anger	−0.520 ***	0.526 ***	0.365 *	0.274	0.406 ***	0.762 ***
Disgust	−0.587 ***	0.655 ***	0.667 **	0.429 ***	0.571 ***	0.713 ***
Sadness	−0.506 ***	0.400 *	0.409 ***	0.349 *	0.290	0.551 ***

* *p* < 0.05, ** *p* < 0.01, *** *p* < 0.001.

**Table 7:** Details of multiple regression—emotions and moral concern/cultural values.

Dependent Variable	Final Independent Variable (s)	*Beta*	*t*	*R^2^(* *△R^2^)*	*F*
Individualism	Disgust	−0.587	−4.470 ***	0.345	19.985 ***
Collectivism	Disgust	0.655	5.340 ***	0.429	28.515 ***
Fairness Vice	Disgust	0.667	5.520 ***	0.445	30.468 ***
Purity Vice (Ridge parameter *K* = 0.048)	Anger	0.307	2.783 ***		
	Happiness	−0.451	−4.032 ***		
	Disgust	0.256	2.480 *	0.885	43.282 ***
In-group Vice	Disgust	0.429	2.927 ***	0.184	8.568 ***
Authority Virtue	Disgust	0.571	4.286 ***	0.326	18.366 ***

* *p* < 0.05, *** *p* < 0.001.

**Table 8:** Correlation coefficients between emotions and behavioral intentions.

Emotion	Protection Intention	Isolation Intention	Anti-Disease Intention	Aid Intention
Happiness	−0.649 ***	−0.483 ***	−0.575 ***	−0.540 ***
Anger	0.452 ***	0.527 ***	0.521 ***	0.671 ***
Disgust	0.449 ***	0.679 ***	0.647 ***	0.610 ***
Sadness	0.209	0.515 ***	0.623 ***	0.557 ***

*** *p* < 0.001.

**Table 9:** Details of multiple regression—emotions and behavioral intentions.

Dependent Variable	Final Independent Variable (s)	*Beta*	*t*	*R^2^*( △*R^2^*)	*F*
Protection Intention	Happiness	−0.649	−5.258 ***	0.421	27.650 ***
Isolation Intention	Disgust	0.679	5.699 ***	0.461	32.479 ***
Aid Intention (Ridge parameter *K* = 0.061)	Sadness	0.335	2.743 ***		
	Anger	0.531	4.343 ***	0.737	22.013 ***
Anti-Disease Intention (Ridge parameter *K* = 0.048)	Sadness	0.413	3.348 ***		
	Disgust	0.458	3.708 ***	0.744	22.993 ***

*** *p* < 0.001.

**Table 10:** Correlation coefficients between moral concern/cultural values and behavioral intentions.

Moral Concern/Collectivism/Individualism	Protection Intention	Isolation Intention	Anti-Disease Intention
Individualism	−0.614 ***	−0.708 ***	−0.729 ***
Collectivism	0.402 ***	0.567 ***	0.611 ***
Fairness Vice	0.669 ***	0.765 ***	0.782 ***
Purity Vice	0.787 ***	0.764 ***	0.791 ***
Authority Virtue	0.670 ***	0.526 ***	0.502 ***
In-group Vice	0.223	0.429 ***	0.457 ***

*** *p* < 0.001.

**Table 11:** Details of multiple regression—moral concern/cultural values and behavioral intentions.

Dependent Variable	Final Independent Variable (s)	*Beta*	*t*	*R^2^(* *△R^2^)*	*F*
Protection Intention (Ridge parameter *K* = 0.042)	Purity Vice	0.620	4.607 ***		
	Authority Virtue	0.242	1.799 ***	0.806	34.362 ***
Isolation Intention (Ridge parameter *K* = 0.040)	Purity Vice	0.452	3.570 ***		
	Fairness Vice	0.452	3.574 ***	0.831	41.363 ***
Anti-Disease Intention (Ridge parameter *K* = 0.032)	Purity Vice	0.272	2.288 *		
	Fairness Vice	0.305	2.742 ***		
	Individualism	0.417	3.497 ***	0.894	47.813 ***

*** *p* < 0.001.

**Table 12:** Details of mediation analysis.

	Estimate	95% CI Lower	95% CI Upper	*p*
**Disgust–Purity Vice–Protection Intention**				
**ACME**	0.674	0.483	0.86	<0.01
**ADE**	−0.222	−0.431	0.04	0.08
**Total Effect**	0.452	0.220	0.69	<0.01
**Disgust–Purity Vice–Anti-Disease Intention**				
**ACME**	0.2724	0.1271	0.42	<0.01
**ADE**	0.0789	−0.0457	0.21	0.36
**Total Effect**	0.3513	0.1924	0.46	<0.01
**Disgust–Purity Vice–Isolation Intention**				
**ACME**	0.008211	0.004236	0.01	<0.01
**ADE**	0.005214	−0.000313	0.01	0.08
**Total Effect**	0.013425	0.008578	0.02	<0.01
**Disgust–Authority Virtue–Protection Intention**				
**ACME**	0.3789	0.2255	0.57	<0.01
**ADE**	0.0921	−0.1746	0.38	0.36
**Total Effect**	0.4710	0.1532	0.72	<0.01
**Disgust–Fairness Vice–Anti-Disease Intention**				
**ACME**	0.234	0.126	0.38	<0.01
**ADE**	0.124	−0.027	0.21	0.16
**Total Effect**	0.358	0.220	0.51	<0.01
**Disgust–Fairness Vice–Isolation Intention**				
**ACME**	0.007337	0.004451	0.01	<0.01
**ADE**	0.005981	0.000236	0.01	0.08
**Total Effect**	0.013318	0.009348	0.02	<0.01
**Disgust–Individualism–Anti-Disease Intention**				
**ACME**	0.224	0.125	0.34	<0.01
**ADE**	0.113	−0.016	0.21	0.04
**Total Effect**	0.337	0.233	0.43	<0.01

ACME: Average causal mediation effects, namely, indirect effect. ADE: Average direct effects, namely, direct effect.

## Data Availability

All relevant data are within the paper and its Supplementary Materials files.

## Supplementary Materials

Supplementary materials can be found at https://www.mdpi.com/1660-4601/17/23/8766/s1. File S1. Details of the Dictionaries (XLSX); File S2. Raw Data. (XLSX); File S3. Details of Regression Models (DOCX); File S4. Processes of multiple regression (DOCX).
