# Supplementary material for: Exploring the Mechanisms of Influence on COVID-19 Preventive Behaviors in China’s Social Media Users
Source: Int J Environ Res Public Health. 2020 Nov 25;17(23):8766. doi: 10.3390/ijerph17238766 (PMC7728355; doi:10.3390/ijerph17238766)
Supplement: Supplementary file 1 [file ijerph-17-08766-s001.zip › S3 File∩╝ÜDetails of Regression Models.docx]

| **Dependent variable** | ***Beta*** | ***t*** | ***R^2^*** | ***F*** |
| --- | --- | --- | --- | --- |
| **Individualism** | -0.730 | -6.575^***^ | 0.532 | 43.236^***^ |
| **Collectivism** | 0.532 | 3.872^***^ | 0.283 | 14.995^***^ |
| **Harm Virtue** | 0.030 | 0.186 | 0.001 | 0.035 |
| **Harm Vice** | 0.266 | 1.702 | 0.071 | 2.896 |
| **Fairness Virtue** | 0.218 | 1.379 | 0.048 | 1.901 |
| **Fairness Vice** | 0.605 | 4.689^***^ | 0.367 | 21.897^***^ |
| **Purity Virtue** | 0.010 | 0.062 | 0.000 | 0.004 |
| **Purity Vice** | 0.646 | 5.211^***^ | 0.417 | 27.155^***^ |
| **In-group Virtue** | -0.382 | -2.551 | 0.146 | 6.509 |
| **In-group Vice** | 0.321 | 2.091^*^ | 0.103 | 4.371^*^ |
| **Authority Virtue** | 0.334 | 2.184^*^ | 0.112 | 4.769^*^ |
| **Authority Vice** | 0.032 | 0.198 | 0.001 | 0.039 |
| **Happiness** | -0.456 | -3.155^***^ | 0.208 | 9.952^***^ |
| **Disgust** | 0.524 | 3.795^***^ | 0.275 | 14.404^***^ |
| **Sadness** | 0.568 | 4.259^***^ | 0.323 | 18.142^***^ |
| **Fear** | 0.163 | 1.019 | 0.027 | 1.039 |
| **Anger** | 0.454 | 3.141^**^ | 0.206 | 9.868^**^ |
| **Protection Intention** | 0.363 | 2.403^*^ | 0.132 | 5.777^*^ |
| **Isolation Intention** | 0.707 | 6.167^***^ | 0.500 | 38.030^***^ |
| **Aid Intention** | 0.565 | 4.220^***^ | 0.319 | 17.810^***^ |
| **Anti-Disease Intention** | 0.893 | 12.199^***^ | 0.797 | 148.826^***^ |
| **Dispelling Rumors Intention** | 0.014 | 0.086 | 0.000 | 0.007 |

^*^ *p* < 0.05, ^**^ *p* < 0.01, ^***^ *p* < 0.001

The independent variable of each regression model was total confirmed cases.
