# Supplementary material for: Exploring the Mechanisms of Influence on COVID-19 Preventive Behaviors in China’s Social Media Users
Source: Int J Environ Res Public Health. 2020 Nov 25;17(23):8766. doi: 10.3390/ijerph17238766 (PMC7728355; doi:10.3390/ijerph17238766)
Supplement: Supplementary file 1 [file ijerph-17-08766-s001.zip › S4 File∩╝ÜProcesses of multiple regression.docx]

| **Dependent variable** | **Independent variables** | ***Beta*** | ***t*** | ***△R^2^*** | ***F*** |
| --- | --- | --- | --- | --- | --- |
| **Individualism** **(Ridge parameter *K* = 0.185)** | Disgust | -0.275 | -2.260^*^ | - | - |
|  | Anger | -0.163 | -1.332 | - | - |
|  | Happiness | 0.111 | 0.884 | - | - |
|  | Sadness | -0.211 | -1.801 | 0.377 | 6.893^***^ |
| **Collectivism (Ridge parameter *K* = 0.154)** | Disgust | 0.376 | 3.049^***^ | - | - |
|  | Anger | 0.128 | 1.027 | - | - |
|  | Happiness | -0.195 | -1.517 | - | - |
|  | Sadness | 0.058 | 0.489 | 0.412 | 8.029^***^ |
| **Fairness Vice (Ridge parameter *K* = 0.275)** | Disgust | 0.436 | 4.068^***^ | - | - |
|  | Anger | -0.010 | -0.091 | - | - |
|  | Happiness | -0.099 | -0.916 | - | - |
|  | Sadness | 0.025 | 0.124 | 0.378 | 6.931^***^ |
| **Purity Vice** **(Ridge parameter *K* = 0.056)** | Disgust | 0.240 | 2.561^*^ | - | - |
|  | Anger | 0.301 | 3.114^**^ | - | - |
|  | Happiness | -0.391 | -3.823^***^ | - | - |
|  | Sadness | 0.093 | 1.070 | 0.763 | 32.310^***^ |
| **In-group Vice (Ridge parameter *K* = 0.143)** | Sadness | 0.184 | 1.360 | - | - |
|  | Disgust | 0.301 | 2.224^*^ | 0.169 | 4.960^*^ |
| **Authority Virtue (Ridge parameter *K* = 0.157)** | Disgust | 0.351 | 2.662^*^ | - | - |
|  | Anger | 0.029 | 0.218 | - | - |
|  | Happiness | -0.243 | -1.803 | 0.313 | 6.919^***^ |

***Emotions and cognitive processes:***

The results of the first multiple regression

^*^ *p* < 0.05, ^**^ *p* < 0.01, ^***^ *p* < 0.001.

The results of the second multiple regression were shown in manuscript

***Emotions and preventive behavioral intentions:***

The results of the first multiple regression

| **Dependent variable** | **Independent variables** | ***Beta*** | ***t*** | ***△R^2^*** | ***F*** |
| --- | --- | --- | --- | --- | --- |
| **Protection Intention (Ridge parameter *K* = 0.190)** | Happiness | -0.447 | -3.657^***^ | - | - |
|  | Disgust | 0.114 | 0.944 | - | - |
|  | Anger | 0.071 | 0.582 | 0.366 | 8.507^***^ |
| **Isolation Intention (Ridge parameter *K* = 0.129)** | Disgust | 0.430 | 3.497^***^ | - | - |
|  | Happiness | 0.012 | 0.092 | - | - |
|  | Anger | 0.162 | 1.306 | - | - |
|  | Sadness | 0.227 | 1.948 | 0.468 | 9.573^***^ |
| **Aid Intention (Ridge parameter *K* = 0.190)** | Disgust | 0.231 | 2.183^*^ | - | ^-^ |
|  | Anger | 0.348 | 3.264^**^ | - | - |
|  | Happiness | -0.025 | -0.227 | - | - |
|  | Sadness | 0.245 | 2.406^*^ | 0.521 | 11.587^***^ |
| **Anti-Disease Intention (Ridge parameter *K* = 0.124)** | Disgust | 0.329 | 2.773^**^ | - | - |
|  | Happiness | -0.111 | -0.890 | - | - |
|  | Anger | 0.101 | 0.840 | - | - |
|  | Sadness | 0.328 | 2.922^**^ | 0.513 | 11.287^***^ |

^*^ *p* < 0.05, ^**^ *p* < 0.01, ^***^ *p* < 0.001.

The results of the second multiple regression

| **Dependent variable** | **Independent variables** | ***Beta*** | ***t*** | ***△R^2^*** | ***F*** |
| --- | --- | --- | --- | --- | --- |
| **Aid Intention (Ridge parameter *K* = 0.079)** | Sadness | 0.265 | 2.376^*^ | - | - |
|  | Disgust | 0.239 | 1.984 | - | - |
|  | Anger | 0.388 | 3.263^**^ | 0.540 | 16.272^***^ |

^*^ *p* < 0.05, ^**^ *p* < 0.01, ^***^ *p* < 0.001.

The results of the third multiple regression were shown in manuscript

***Cognitive processes and preventive behavioral intentions:***

The results of the first multiple regression

| **Dependent variable** | **Independent variables** | ***Beta*** | ***t*** | ***R^2^(△R^2^)*** | ***F*** |
| --- | --- | --- | --- | --- | --- |
| **Protection Intention (Ridge parameter *K* = 0.075)** | Purity Vice | 0.484 | 3.781^***^ | - | - |
|  | Authority Virtue | 0.244 | 2.099^**^ | - | - |
|  | Individualism | -0.113 | -0.838 | - | - |
|  | Collectivism | -0.201 | -1.665 | - | - |
|  | Fairness Vice | 0.201 | 1.705 | 0.649 | 15.393^***^ |
| **Isolation Intention (Ridge parameter *K* = 0.088)** | Purity Vice | 0.369 | 3.010^**^ | - | - |
|  | Fairness Vice | 0.380 | 3.205^**^ | - | - |
|  | Individualism | -0.171 | -1.334 | - | - |
|  | Collectivism | 0.049 | 0.416 | - | - |
|  | Authority Virtue | -0.084 | -0.744 | - | - |
|  | In-group Vice | 0.003 | 0.032 | 0.654 | 13.267^***^ |
| **Anti-Disease Intention (Ridge parameter *K* = 0.141)** | Purity Vice | 0.324 | 3.861^***^ | - | - |
|  | Fairness Vice | 0.309 | 3.778^***^ | - | - |
|  | Individualism | -0.385 | -4.526^***^ | - | - |
|  | Collectivism | -0.019 | -0.235 | - | - |
|  | Authority Virtue | -0.144 | -1.822 | - | - |
|  | In-group Vice | -0.027 | -0.370 | 0.788 | 25.206^***^ |

^*^ *p* < 0.05, ^**^ *p* < 0.01, ^***^ *p* < 0.001.

The results of the second multiple regression were shown in manuscript
